# The clinical importance and correlations of post-partum changes in the clinical findings, reproductive cyclicity, serum-milk oxidant/antioxidant parameters as a stress indicator in female dromedary camel (*Camelus dromedarius*) and their effect on milk palatability

**DOI:** 10.1007/s11259-024-10335-x

**Published:** 2024-02-27

**Authors:** Arafat Khalphallah, Abdulaziz H. Almuhanna, Taher Al-Daek, Abdulrahman Alhaider, Enas Elmeligy, Ragab H. Mohamed, Abdulrahman Abdulkarim, Marwa I. Khalifa, Shefaa M. Bazeed, Khaled A. Khesruf, Rezk Said Ghallab, Asem M. Zakaria

**Affiliations:** 1 Division of Internal Medicine, Department of Animal Medicine, Faculty of Veterinary Medicine, Assiut University, Assiut, 71526 Egypt; 2 Department of clinical studies, Collage of Veterinary Medicine, King Faisal University, Al-Ahsa, Saudi Arabia; 3 Faculty of Veterinary Medicine, Omar Al-Mukhtar University, 919, Al-bayda, Libya; 4 Veterinary Teaching Hospital, Faculty of Veterinary Medicine, Assiut University, Assiut, 71526 Egypt; 5 Department of Theriogenology, Obstetrics, and Artificial Insemination, Faculty of Veterinary Medicine, Aswan University, Aswan, 81528 Egypt; 6 Department of Food Hygiene, Faculty of Veterinary Medicine, Aswan University, Aswan, 81528 Egypt; 7 Department of Biochemistry and Animal Physiology, Faculty of Veterinary Medicine, Badr University in Cairo (BUC), Cairo, Egypt; 8 Department of Animal diseases, Faculty of Veterinary Medicine, Aleppo University, Aleppo, Syria; 9 Department of Theriogenology, Faculty of Veterinary Medicine, Matrouh University, Matrouh, 51744 Egypt

**Keywords:** Cortisol, Serum or milk oxidants/antioxidant biomarkers, Stress indicators, She-camel fertility, Steroids hormones

## Abstract

Dramatic metabolic changes during pregnancy and post-partum period resulted in alteration of the biochemical parameters in dromedary she-camels. The current study focused on assessment of stress indicators in post-partum dromedary she-camels on days 14, 28 and 42 post-calving through monitoring the clinical findings, serum steroid hormones, serum or milk oxidant/antioxidant indicators, and milk somatic cell count (SCC) status with reference to serum lipid profile changes. The study also stated several correlations between reproductive cyclicity parameters, stress biomarkers and serum-milk oxidant/antioxidant indicators. The study was conducted on clinically healthy recently calved she-camels (*n* = 25). They were subjected to clinical and laboratory assays including lipid profiles, serum steroid hormones [Progesterone (P4) and estradiol (E2)], serum or milk oxidant/antioxidant biomarkers [Malondialdehyde (MDA), reduced glutathione (GSH) and cortisol], and milk SCC on days 14, 21 and 28 post-calving. The study concluded the influence of stress as a result of lactation in post-partum period in recently calved she-camels and its relationship with reproductive cyclicity as well as changes in serum steroids, lipid profiles, serum-milk oxidant/antioxidants parameters, and milk SCC that was reflected through significant elevations in serum levels of P4, E2, cortisol, MDA and glucose, and milk values of MDA, cortisol and SCC as well as significant drop in serum levels of GSH, TPs, albumins and globulins on day 14 post-calving comparing with their values particularly on day 42. The study stated variable correlation relationships between reproductive cyclicity parameters, lipid profiles, serum-milk oxidant/antioxidants parameters and milk SCC.

## Introduction

Arabian camels were well accommodated to the severe desert environment conditions which might be owned to their due to their great metabolic and physiological adaptation (Seboussi et al. 2009).

Dromedary camels were highly accommodated to extremely exaggerated environments in wide range of tropical areas, semi-arid and arid areas in Africa, Asia, and Australia (Gaughan 2011; Faye 2015; Tibary and El Allali 2020). These one-humped camels were highly important as multipurpose animals including milk, meat, race, and working purposes (Marai et al. 2009).

Dromedary camels had lower reproductive capability as this was probably contributed to seasonality, prolonged inter-calving periods (Tibary and El Allali 2020). They were known as short-day seasonal breeders whereas the reproduction cyclicity was disturbed through summer season (Marai et al. 2009), because of high summer temperature as a main factor that reduced reproductive cyclicity during this low breeding season (Marai et al. 2009).

Oxidative stress had been identified as the condition in which there was insufficient antioxidants to counter the overproduction of oxidants such as reactive oxygen species (ROS) or free radicals (Agarwal et al. 2005). Increased oxygen requirements occurred during physiological stress (Gitto et al. 2002), which in turn, stimulated the overproduction free radicals or ROS. The oxidative destruction of lipid or what was called lipid peroxidation considered one of important output of oxidative stress that induced releasing malondialdehyde (MDA). MDA was a stable end-product or free radicals in comparison to other short lifespan free radicals (Sharma et al. 2011). Overproduction of ROS was controlled by antioxidants defense mechanisms. These antioxidants defense mechanisms were enhanced by antioxidant enzymes (Trevisan et al. 2001).

MDA as enzymatic antioxidants were widely studied to assess oxidant stress (Celi 2010). Oxidative stress was reported within many diseased status, such as enteritis, mastitis, sepsis, diseases of respiratory system and joint disease. It also was described during transport and in pregnancy in ruminants. Moreover, oxidative stress might be reported for the efficient treatments and prognosis of these status (Piccione et al. 2013).

Reduced glutathione (GSH) was the most natural low molecular weight tripeptide antioxidant. It played its antioxidant role as a scavenger of ROS. Moreover, GSH played a pro-oxidant role, which had a lesser efficacy than its antioxidant role (Gaucher et al. 2018). On other side, catalase (CAT) considered as one of the first line defense antioxidant enzymes. Long-term oxidative stress oftenly caused continuous elevation in MDA and clear drop in the antioxidants levels including mainly antioxidant enzymes such as CAT and GSH, after transient increase to counter and alleviate the toxic harmful effects of oxidant substances (Kale et al. 1999; Valko et al. 2007; Sharma et al. 2011).

Camels’ milk has been the subject of several investigations as a potential protein source for producing bioactive protein hydrolysates with antioxidant properties (Al-Saleh et al. 2014; Shori and Baba 2014). By reducing their shelf life and degrading their nutritional value, oxidative reactions had a negative impact on milk and dairy products (Havemose et al. 2006; Krzyżewski et al. 2012).

Since excessive cortisol was produced and released into the systemic circulation under stressful situations, measuring blood cortisol concentration was utilised as a routine approach for assessing stress in farm animals (Mormède et al. 2007). The main stresses affecting cattle physiology were changes in the environment and in management (Comin et al. 2011). In general, cortisol supported energy consumption, reproduction, immunological response, inflammatory processes, growth, and brain function to assist the body maintain homeostasis. Nevertheless, a sustained increase in glucocorticoid levels had a deleterious impact on immunological response or reproductive activity (Minton 1994; Möstl and Palme 2002).

Somatic cells, such as lymphocytes, macrophages, polymorphonuclear, and certain epithelial cells, were immune system cells that were a component of the body’s natural defense mechanisms (Ruegg and Pantoja 2013).

Milk somatic cell count (SCC) was a measure of the health of the udder that could really discriminate between infected and uninfected quarters. Increased SCC above normal levels, whether in bulk milk or a particular quarter, denoted the presence of inflammation and an affected udder (Pantoja et al. 2009; Sukur and Esendal 2020).

SCC was frequently used in cows to monitor the quality of the milk in the dairy sector, but it was less frequently employed in camels because the physiological fluctuations and basal cell levels in this species were not yet documented (Abdurahmann et al. 1992; Nagy et al. 2013), which made interpretation difficult. Notably, there were few studies done on lactation variance (Nagy et al. 2013; Saleh et al. 2013; Kaskous 2016).

Monitoring the clinical changes and studying the effect of lactation as an efficacious stress factor in the post-calving she-camel and their relationships with reproductive cyclicity and serum oxidant-antioxidant status as well as milk palatability, milk SCC and milk oxidant/antioxidant status of the she-camels, needed further analysis. Accordingly, the current study focused on assessment of stress indicators in post-calving dromedary she-camels on days 14, 28 and 42 through monitoring the changes in clinical findings, serum steroid hormones, serum or milk oxidant/antioxidant indicators, and milk SCC status with reference to serum lipid profile changes. The study also stated several correlations between reproductive cyclicity parameters i.e. serum progesterone (P4) and serum estradiol (E2), stress biomarkers and serum-milk oxidant/antioxidant indicators.

## Materials and methods

### Animals

25 post-calving female camels that were raised in private farms in the Aswan governorates of Egypt. The investigated she-camels were taken kindly from the farm by a permission that was taken from the farm owner. Their ages ranged from 10 to 12 years, and their body weights were between 600 and 750 kg. Camels were kept in a spacious yard. All animals were raised in identical corrals in a 1300 m^2^ open yard under identical conditions. There was more than 20 m^2^ of space allotted for each head. The majority of the diet given to the animals in groups consisted of commercial concentrates mixtures (12% crude protein and 70% total digestible nutrients; TDN) (4 kg per head per day), along with roughly 10 kg per day of roughage material, which was Egyptian clover during the winter and Egyptian clover hay during the summer (10 kg per head per day). All the day long, water was accessible. These animals undergone clinical and laboratory examinations to ensure they were free of any pathological issues. On days 14, 28, and 42 post-partum, she-camels underwent clinical examinations and laboratory samples were taken.

### Samples

The jugular vein was used to collect whole blood and serum samples, and all necessary steps were done during sample preparation and collection to ensure a precise evaluation of haematological and biochemical indices. For subsequent hormonal studies using commercial test kits in accordance with the usual supplier instructions, serum samples were obtained and stored frozen at -20 °C (Coles 1986). The investigated camels were sampled on days 14, 28, and 42 post-partum for laboratory tests.

On days 14, 28, and 42 post-partum, milk samples were collected during routine morning milking into sterile screw-capped bottles and were immediately transported to the laboratory where they were stored overnight at 4 ^◦^C before analysis or kept frozen until they were examined for milk MDA, cortisol, and SCC (Hamed et al. 2010).

### Clinical examination

On days 14, 28, and 42 post-partum, the clinical examinations included measurements of heart and respiratory rates as well as the recording of rectal temperatures and rumen movements according to the methods described by Fowler (2010); Abdel-Rahman et al. (2017); Hassan et al. (2019); Mohamed et al. (2021).

### Complete blood picture indices

Red blood corpuscles (RBCs), haemoglobin (Hb), total leucocytic count (TLC), and packed cell volume (PCV) were manually measured according to methods described by Coles (1986); Harvey (2001); Latimer et al. (2011); Faye and Bengoumi (2018).

### Hormonal analysis

Serum P4 values were estimated using Oxford Biomedical Research commercial kits (Rochester Hills, Michigan, USA) and by enzyme-linked immunosorbent assay (ELISA-Sandwich Protocol). Commercial radioimmunoassay kits (Parameters commercial kits, Inc. 614 McKinley Place NE Minneapolis, Toll Free USA, Canada) were used to estimate serum cortisol and E2 concentrations of cortisol and E2.

### Serum oxidant and antioxidants biomarkers assays

Serum MDA values as lipid peroxidation parameters (Biodiagnostic commercial kits, Cairo, Egypt) and serum concentrations of antioxidants as biomarkers (Biodiagnostic assay kits, Cairo, Egypt), including GSH, were measured using the Spectro Ultraviolet-Vis RS spectrophotometer (Labomed, Inc., Los Angeles, CA, USA).

### Serum lipid Profile Indices

The Spectro Ultraviolet-Vis RS spectrophotometer (Labomed, Inc., Los Angeles, CA, USA) was used to measure blood levels of total proteins (TP), albumins and glucose. Serum globulins were estimated by subtraction of albumins from TP. Gamma Trade Company (Cairo, Egypt) provided kits for plasma glucose while and BioMed (Cairo, Egypt) provided commercial kits for TP and albumins.

### The palatability of milk

The palatability of milk was determined across 42 days of parturition in camel by a group from nine qualified paleness, adopted to consume camel milk regularly. They test milk for color, flavor, taste and over all acceptability using a hedonic scale graduated from 1 to 5 according to Khalifa and Zakaria (2019).

### Milk oxidants/antioxidants biomarkers assays

Milk MDA values were measured using thiobarbituric acid (TBA) method according to Ali et al. (2016). According to Gellrich et al. (2015), a highly sensitive competitive ELISA was used to estimate milk cortisol concentrations.

### Milk SCC

Somatic cell counter was the suitable technique to automatically measures milk SCC (Kamal et al. 2014).

### Statistical analysis

SPSS statistical software program for Windows (version10.0.1, SPSS Inc., Chicago, IL., USA) was used for data analysis. The reported obtained data were expressed as mean ± SD. The data of clinical parameters and laboratory assays were analyzed by general linear model repeated measures ANOVA whereas significance level was set at *p* < 0.05. The significance of differences was assessed between the means at various sampling periods i.e. days 14, 28 and 42. Correlation coefficient was calculated using Pearson Correlation at *p* < 0.05 or *p* < 0.01 between serum steroids hormones, serum lipid profile indices, serum oxidant/antioxidant indicators (Stress indicators) i.e. MDA and GSH, milk oxidant/antioxidant indicators (Stress indicators) i.e. MDA and cortisol, and milk SCC in clinically healthy post-calving she-camels.

## Results

### Clinical findings and blood picture indices

The clinical findings in post-calving she-camels were within the reference ranges whereas no significant changes were stated between days 14, 28 and 42 post-partum either for temperature, pulse, respirations or for rumen motility (Table 1).

**Table 1:** Mean values (**M ± SD**) of temperature, pulse, respiration and rumen movements in clinically healthy post-calved she-camels (*n* = 25)

	Days post-partum			Reference values
	Day 14	Day 28	Day 42	
**Temperature** (°C)	37.81 ± 0.22^a^	37.66 ± 0.43^a^	37.86 ± 0.41^a^	(37.52 ± 0.09)^1^ or (37.2 ± 0.77)^2^
**Pulse** (Beats/min)	28.06 ± 2.66^a^	29.33 ± 2.09^a^	30.11 ± 1.88^a^	(32–36)^3^ or (24–48/min)^4^
**Respiration** (Breaths/min)	12.74 ± 2.17^a^	11.28 ± 1.87^a^	12.86 ± 2.11^a^	(12.55 ± 0.30)^1^ or (8–18)^5^
**Rumen motility** (Movements/2min)	3.15 ± 0.39^a^	2.94 ± 0.44^a^	3.12 ± 0.67^a^	(4.25 ± 0.14)^1^ or (4.3 ± 0.14)^6^

^a^Means within the same row with different superscript letters in different sampling days were significantly different (*P* < 0.05). Reference values according to Hassan et al. (2019)^1^; Hamad et al. (2017)^2^; Fowler (2010)^3^; Bhatt et al. (1960)^4^; Nielsen (1964)^5^; Kamr et al. (2020)^6^

Whole blood picture indices i.e. RBCs, Hb, PCV and TLC reported no significant changes between days 14, 28 and 42 post-partum as they were within the reference ranges (Table 2).

**Table 2:** Mean values (M ± SD) of whole blood picture indices in in clinically healthy post-calved she-camels (*n* = 25)

	Days post-partum			Reference values
	Day 14	Day 28	Day 42	
**RBCs** (_X_10^12^/L)	9.53 ± 2.06^a^	9.74 ± 1.74^a^	10.11 ± 2.22^a^	(7.5–12)^1^
**Hb** (g/L)	126.13 ± 4.33^a^	131 ± 3.82^a^	128.77 ± 4.02^a^	(120–150)^1^
**PCV** (L/L)	0.32 ± 0.06^a^	0.31 ± 0.03^a^	0.33 ± 0.04^a^	(0.26–0.38)^1^
**TLC** (_X_10^9^/L)	9.78 ± 1.88^a^	9.84 ± 2.01^a^	10.03 ± 1.85^a^	(6-13.5)^1^

RBCs: Red blood corpuscles. Hb: Haemoglobin. PCV: Packed cell volume. TLC: Total leucocytic count. ^a^Means within the same row with different superscript letters in different sampling days were significantly different (*P* < 0.05). Reference values according to Fowler (2010)^1^

### Steroids hormones

Serum concentrations of P4 were significantly (*p* < 0.05) decreased on days 28 and 42 post-calving in she-camels comparing with P4 values of day 14. Serum values of P4 were higher than their reference ranges throughout the current study (Table 3).

**Table 3:** Mean values (**M ± SD**) of serum steroids hormones i.e. P4, E2 and cortisol, MDA and GSH in clinically healthy post-calved she-camels (*n* = 25)

	Days post-partum			Reference values
	Day 14	Day 28	Day 42	
**P4** (ng/mL)	2.46 ± 0.47^a^	2.13 ± 0.49^b^	2.03 ± 0.42^c^	(0.93 ± 0.21)^1^ or (0.29 ± 0.26–0.33 ± 0.44)^2^
**E2** (pg/mL)	133.56 ± 23.41^a^	115.33 ± 23.49^b^	131.78 ± 27.99^a^	(180.38 ± 50.61)^1^ or (1.29 ± 1.44–2.66 ± 1.98)^2^
**Cortisol** (nmol/L)	88.28 ± 23.29^a^	50.76 ± 9.83^b^	39.33 ± 10.07^c^	(38.17 ± 3.99)^3^
**MDA** (µmol/L)	14.36 ± 0.81^a^	11.08 ± 0.88^b^	9.11 ± 0.64^c^	(13.2 ± 0.6)^4^ or (13.89 ± 0.94)^5^ or (10·23 − 11·62)^6^
**GSH** (mg/dL)	29.05 ± 1.15^c^	31.75 ± 1.42^b^	35.53 ± 1.02^a^	(37.68 ± 2.35)^1^

P4: Progesterone. E2: Estradiol. MDA: Malondialdehyde. GSH: Reduced glutathione. ^a−c^Means within the same row with different superscript letters in different sampling days were significantly different (*P* < 0.05). Reference values according to Mohamed et al. (2021)^1^; Ayoub et al. (2003)^2^; Saeb et al. (2010)^3^, El-Bahr and El-Deeb (2016)^4^; Saleh et al. (2009)^5^; El-Deeb and Elmoslemany (2015)^6^

Post calving, she-camels had a significant (*p* < 0.05) drop in their serum concentrations of E2 on day 28 after parturition when they compared with their values on day 14. Soon later, serum E2 values were significantly (*p* < 0.05) elevated on day 28. Their serum concentrations were within their reference ranges throughout the present study (Table 3).

Serum levels of cortisol in recently calving she-camels were significantly (*p* < 0.05) reduced on days 28 and 42 comparing with E2 values on day 14. Serum levels of cortisol were higher than their reference ranges throughout the current study (Table 3).

### Serum Oxidants/Antioxidants biomarkers

Regarding oxidants biomarkers, serum MDA values were significantly higher (*p* < 0.05) in on day 14 then significantly (*p* < 0.05) reduced soon later on days 28 and 42 in post-partum she-camels. Serum MDA values were higher than their reference values on days 14 and became within the reference ranges on days 28 and 42 (Table 3).

In she-camels, serum levels of GSH on days 28 and 42 post-calving were significantly higher than their values on day 14. Serum concentrations of GSH had greater values than their reference ranges on days 14 and 28 post-calving while they reached their reference range on day 42 (Table 3).

### Serum lipid profiles indicators

Serum lipid parameters showed significant changes whereas some of them i.e. glucose were significantly (*p* < 0.05) higher while some of them i.e. TPs, albumins and globulins, were significantly (*p* < 0.05) lower on day 14 when they compared with their values on days 28–42 in post-partum she-camels. Serum concentrations of glucose, TPs, albumins and globulins were within the reference ranges (Table 4).

**Table 4:** Mean values (**M ± SD**) of serum lipid profiles indices in clinically healthy post-calved she-camels (*n* = 25)

	Days post-partum			Reference values
	Day 14	Day 28	Day 42	
**Glucose** (mmol/l)	9.60 ± 0.80^a^	8.86 ± 0.62^c^	9.18 ± 0.64^b^	(6.36 ± 0.35)^1^ or (4.88–6.97)^2^ or (5.01–8.03)^3^
**Total proteins** (g/L)	58.68 ± 2.14^b^	65.99 ± 4.27^a^	66.53 ± 3.31^a^	(61.20 ± 4.30)^1^ or (59.7-106.7)^3^ or (53–78)^4^
**Albumins** (g/L)	26.70 ± 1.26^b^	28.74 ± 2.09^a^	29.00 ± 2.40^a^	(38.30 ± 2.10)^1^ or (30.80 ± 1.38)^2^ or (12.2–77.5)^3^
**Globulins** (g/L)	31.98 ± 1.29^b^	37.24 ± 2.98^a^	37.53 ± 2.72^a^	(18.42–23.87)^2^ or (16–29)^5^

^a−c^Means within the same row with different superscript letters in different sampling days were significantly different (*P* < 0.05). Reference values according to Saeb et al. (2010)^1^; Abdalmula et al. (2019)^2^; Islam et al. (2019)^3^; Mohamed and Hussein (1999)^4^; Kaneko et al. (2008)^5^

### The palatability of milk

**Fig. 1:**
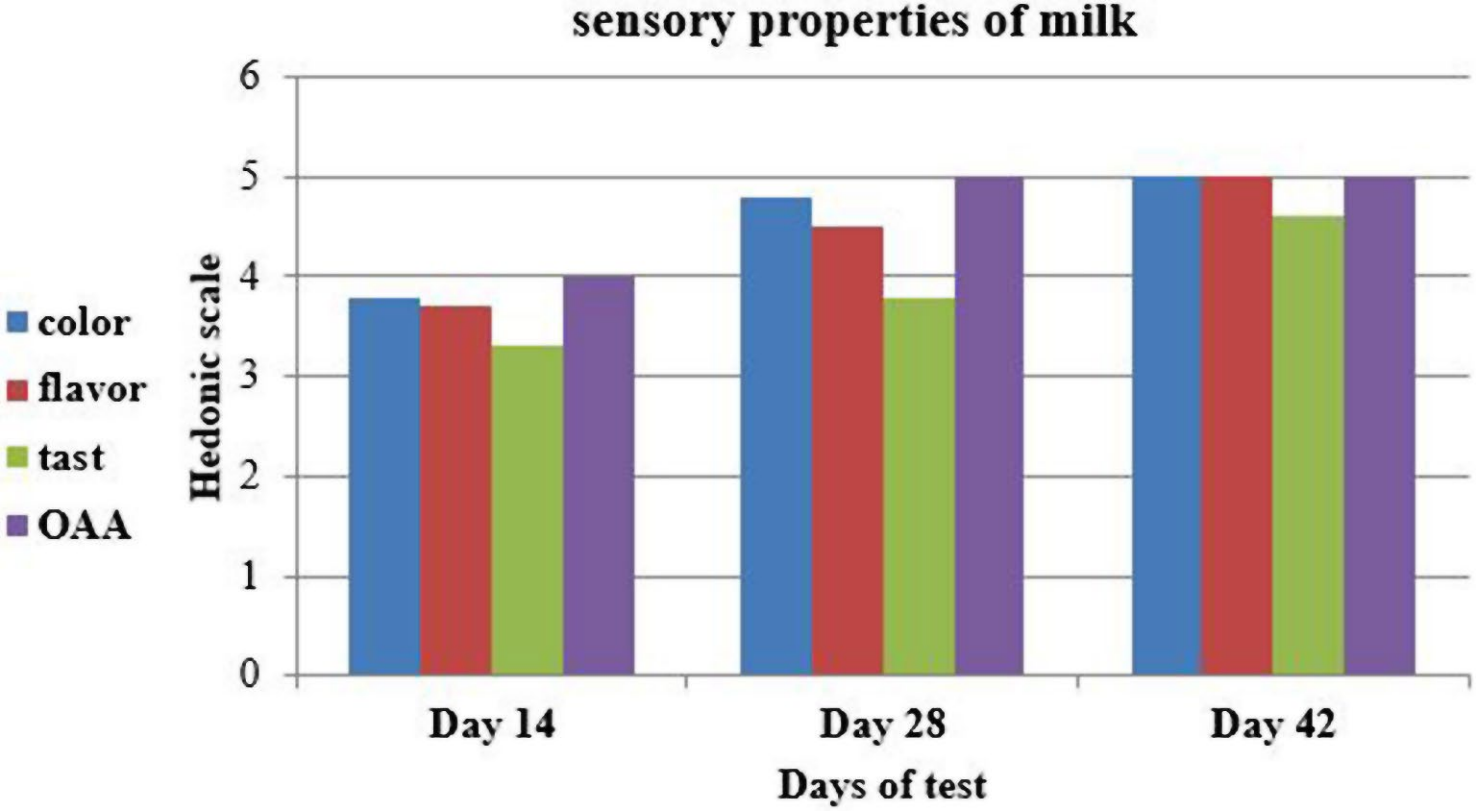
Sensory properties of camel milk across 42 days of parturition. OAA = over all acceptability

The results reveled in Fig. 1. showed that the sensory properties of milk were improving over time. The color on day 14 seemed a little unfamiliar, then it improved on day 28 and became very normal on day 42, might be due to the disappearance of the dark-colored colostrum proteins. Likewise, the smell and taste improved and reached the usual limits on day 42, although the taste of natural camel milk was characterized by some salinity due to the high chlorine content in it, and also affected by postpartum hormones.

### **Milk oxidant/antioxidant biomarkers i.e. MDA and cortisol assays**

Milk values of MDA as milk oxidant indicators were significantly (*p* < 0.05) dropped ^on d^ays 28 and 42 post-partum in investigated she-camels comparing to MDA values on day 14. MDA values in she-camels milk were within their reference ranges throughout the current study (Table 5).

**Table 5:** Mean values (**M ± SD**) of MDA, cortisol and SCC in camel’s milk in clinically healthy post-calved she-camels (*n* = 25)

	Days post-partum			Reference values
	Day 14	Day 28	Day 42	
**MDA** (µmol/L)	18.49 ± 0.90^a^	15.69 ± 0.85^b^	12.83 ± 0.96^c^	(18–28)^1^
**Cortisol** (nmol/L)	3.34 ± 0.29^a^	2.76 ± 0.20^b^	2.15 ± 0.36^c^	(0.41–2.01)^2^
**SCC** (_X_ 10^3^ cells/ml)	130.67 ± 3.08^a^	122.89 ± 2.62^b^	110.22 ± 2.68^c^	14.37–53.07 or 15.57-164.11)^3^ or (25.5 ± 16.4)^4^

MDA: Malondialdehyde. SCC: Somatic cell count. ^a−c^Means within the same row with different superscript letters in different sampling days were significantly different (*P* < 0.05). Reference values according to Kapusta et al. (2018)^1^; Nedić et al. (2017)^2^; Saleh et al. (2013)^3^; Hamed et al. (2010)^4^

A Significant drop (*p* < 0.05) in milk concentrations of cortisol as stress indicator was demonstrated on day 28–42 after calving in she-camels comparing with their values on day 14. Milk levels of cortisol was higher than their references value on days 14–42 post-calving (Table 5).

### Milk SCC

On day 14 following calving, milk SCCs were significantly elevated (*p* < 0.05) then remarkably (*p* < 0.05) dropped afterwards on days 28–42 in investigated she-camels. Milk SCCs during the current study were within their reference intervals (Table 5).

### Correlations between serum steroids hormones, serum lipids, serum-milk oxidant/antioxidant indicators and milk SCC in clinically healthy post-calved she-camels

Different correlation relationships were reported between serum steroids hormones, serum lipid profile indices, serum oxidant/antioxidant indicators (Stress indicators i.e. serum MDA and GSH), milk oxidant/antioxidant indicators (Stress indicators i.e. milk MDA and cortisol), and milk SCC in clinically healthy post-calved she-camels (Table 6).

**Table 6:** Pearson correlation coefficient between serum steroids hormones, serum lipid profile indices, serum oxidant/antioxidant indicators (Stress indicators) i.e. MDA and GSH and milk oxidant/antioxidant indicators (Stress indicators) i.e. MDA and cortisol, and milk SCC in clinically healthy post-calved she-camels (*n* = 25)

	Sr. P4	Sr. E2	Sr. Cortisol	Sr. Glucose	Sr. TP	Sr. Albumins	Sr. Globulins	Sr. MDA	Sr. GSH	Milk MDA	Milk Cortisol	Milk SCC
**Sr.** **P4**		*r* = -0.005	*r* = 0.286	*r* = 0.286	*r* = -0.173	*r* = -0.344	*r* = -0.027	*r* = 0.475^*^	*r* = -0.422^*^	*r* = 0.324	*r* = 0.338	*r* = 0.314
		P_v_ = 0. 981	P_v_ = 0.148	P_v_ = 0.149	P_v_ = 0.389	P_v_ = 0.079	P_v_ = 0.894	P_v_ = 0.012	P_v_ = 0.042	P_v_ = 0.099	P_v_ = 0.085	P_v_ = 0.1105
**Sr.** **E2**			*r* = 0.030	*r* = -0.541^**^	P_v_ = -0.296	*r* = 0.087	*r* = -0.466^*^	*r* = 0.185	*r* = -0.031	*r* = 0.201	*r* = 0.240	*r* = 0.110
			P_v_ = 0.883	P_v_ = 0.004	*r* = 0.133	P_v_ = 0.666	P_v_ = 0.014	P_v_ = 0.356	P_v_ = 0.877	P_v_ = 0.314	P_v_ = 0.227	P_v_ = 0.584
**Sr. Cortisol**				*r* = 0.399^*^	P_v_ = -0.671^**^	*r* = -0.396^*^	*r* = -0.688^**^	*r* = 0.754^**^	*r* = -0.672^**^	*r* = 0.746^**^	*r* = 0.773^**^	*r* = 0.745^**^
				P_v_ = 0.039	*r* = 0.0001	P_v_ = 0.041	P_v_= 0.00007	P_v_= 0.00001	P_v_ = 0.0001	P_v_=0.000008	P_v_ = 0.000002	P_v_ = 0.000008
**Sr. Glucose**					P_v_ = -0.169	*r* = -0.307	*r* = -0.045	*r* = 0.261	*r* = -0.178	*r* = 0.130	*r* = 0.087	*r* = 0.052
					*r* = 0.398	P_v_ = 0.120	P_v_ = 0.823	P_v_ = 0.189	P_v_ = 0.374	P_v_ = 0.518	P_v_ = 0.664	P_v_ = 0.796
**Sr.** **TP**						*r* = 0.764^**^	*r* = 0.917^**^	*r* = -0.710^**^	*r* = 0.587^**^	*r* = -0.715^**^	*r* = -0.679^**^	*r* = -0.613^**^
						P_v_ =0.000003	P_v_=1.93 × 10^− 11^	P_v_ =0.00003	P_v_ = 0.001	P_v_ = 0.00002	P_v_ =0.00009	P_v_ =0.0006
**Sr. Albumins**							*r* = 0.442^*^	*r* = -0.497^**^	*r* = 0.409^*^	*r* = -0.474^*^	*r* = -0.371	*r* = -0.352
							P_v_ = 0.021	P_v_ = 0.008	P_v_ = 0.034	P_v_ = 0.013	P_v_ = 0.056	P_v_ = 0.072
**Sr. Globulins**								*r* = -0.679^**^	*r* = 0.563^**^	*r* = -0.002^**^	*r* = -0.714^**^	*r* = -0.634^**^
								P_v_ =0.00009	P_v_ = 0.002	P_v_ =0.000045	P_v_ = 0.000029	P_v_ = 0.0004
**Sr. MDA**									*r* = -0.890^**^	*r* = 0.911^**^	*r* = 0.842^**^	*r* = 0.856^**^
									P_v_=5.01 × 10^− 10^	P_v_=4.3 × 10^− 11^	P_v_ =3.6 × 10^− 8^	P_v_ =1.2 × 10^− 8^
**Sr. GSH**										*r* = -0.859^**^	*r* = -0.850^**^	*r* = -0.844^**^
										P_v_ =9.6 × 10^− 9^	P_v_ =2.05 × 10^− 8^	P_v_ = 3.2 × 10^− 8^
**Milk MDA**											*r* = 0.885^**^	*r* = 0.899^**^
											P_v_=8.8 × 10^− 10^	P_v_ =1.1 × 10^− 10^
**Milk cortisol**												*r* = 0.884^**^
												P_v_ =9.8 × 10^− 10^
**Milk SCC**												

Sr.: Serum. P4: Progesterone. E2: Estradiol. TP: Total proteins. MDA: Malondialdehyde. GSH: Reduced glutathione. SCC: Somatic cell count. r = Correlation. P_v_: P value: ^*^Significant (two-tailed) *p* < 0.05. ^**^Significant (two-tailed) *p* < 0.01. Gray backgrounds referred to correlation between the same parameter e.g. P_4_ and P_4_ where there was no correlation. Diagonal backgrounds referred that this correlation was previously reported in the previous row e.g. correlation between P_4_ and E_2_ was reported in row 1 and so there was no need to repeat it at row 2

No significant correlations were reported between serum steroid hormones (P4, E2 and cortisol) at post-calving period in examined she-camels. Serum P4 values were not correlated with lipid profiles indices i.e. glucose, TPs, albumins and globulins. Serum E2 were negatively correlated with each of serum glucose and globulins while blood cortisol values were correlated positively with glucose and negatively with blood proteins i.e. TPs, albumins and globulins. She-camels at post-partum period (Days14-42) had positive correlations between steroids hormones i.e. P4 and cortisol, and serum MDA (Stress and oxidant indicators), hence, negative correlations were stated between serum GSH (Antioxidant indicators) and these steroid hormones (P4 and cortisol). No correlations were reported between E2 and each of serum MDA and serum GSH. With except for cortisol, no significant correlations were demonstrated between serum steroids i.e. P4 and E2, and milk stress indictors i.e. MDA, cortisol and SCC. Serum cortisol levels were correlated positively with milk MDA, milk cortisol and SCC (Table 6).

### Correlations between serum lipids, serum-milk oxidant/antioxidant indicators and milk SCC in clinically healthy post-calved she-camels

Blood glucose values were not correlated with other lipid profiles indices i.e. TPs, albumins and globulins. They also were not correlated with serum oxidant/antioxidant biomarkers i.e. MDA and GSH, or with milk oxidant/antioxidant biomarkers i.e. MDA, cortisol and SCC. Positive correlations were observed between serum proteins indicators i.e. TPs, albumins and globulins in investigated she-camels at post-partum period (Days 14–42). These serum proteins were correlated positively with serum GSH, while, they were negatively correlated with each of serum MDA, milk MDA, milk cortisol and milk SCC (Table 6).

#### Correlations between serum-milk oxidant/antioxidant indicators and milk SCC in clinically healthy post-calved she-camels

With except for serum GSH, the examined post-calving she-camels (Days 14–42) had positive correlations between serum-milk oxidant/antioxidant parameters and milk SCC. Serum MDA, milk MDA, milk cortisol and milk SCC were correlated positively with each other. Negative correlations were demonstrated between these biomarkers i.e. serum MDA, milk cortisol and milk SCC, and serum GSH values (Table 6).

## Discussion

### Clinical findings and blood picture indices

The clinical findings in post-calving she-camels (Days 14–42) were within the reference ranges reported by Bhatt et al. (1960); Nielsen (1964); Fowler (2010); Hamad et al. (2017); Hassan et al. (2019); Kamr et al. (2020), whereas no significant changes were stated between days 14, 28 and 42 post-partum either for temperature, pulse, respirations or for rumen motility. These results were supported by Mohamed et al. (2021); Khalphallah et al. (2022). Khalphallah et al. (2022) added that the clinical findings showed no remarkable changes between days 14, 21 and 28 either in control or treated post-partum she-camels. Moreover, Mohamed et al. (2021) stated no significant variations for temperature and rumen motility between pregnant she-camels group (PREG), non-pregnant and lactating group (LACT) and non-pregnant and non-lactating she-camels (NPREG). In contrast, pulse and respiration showed remarkable increase in pregnant she-camels group comparing with lactating and non-pregnant she-camels group. No significant changes were reported either for pulse or for respiration between lactating and non-pregnant she-camels.

The current study revealed that the blood picture indices i.e. RBCs, Hb, PCV and TLC reported no significant changes between days 14, 28 and 42 post-partum in she-camels as they were within the reference ranges mentioned by Fowler (2010). These results were supported by Khalphallah et al. (2022). Khalphallah et al. (2022) mentioned no remarkable changes in whole blood parameters between control and treated she-camels at post-calving days14, 21 and 28. No significant changes were also reported between days14, 21 and 28 in control healthy post-partum she-camels.

#### **Serum steroids hormones**

According to Mohammed et al. (2021) who clarified that she-camels’ biochemical, hormonal, oxidant, and antioxidant status varied depending on whether they were pregnant or lactating. The relationship between steroid hormones, oxidants, and antioxidants in she-camels was also documented. On the other hand, in camels grown under conventional conditions, the physiological conditions had more of a biochemical and hormonal effect than a haematological effects (Muhammad et al. 2011). The current work reported remarkable changes in serum steroids hormones i.e. P4, E2 and cortisol in post-calving she-camels through which significant elevations in serum values of P4, E2 and cortisol were detected on day 14 post-calving followed by significant reduction in their serum concentrations on days 28 and 44 in post-partum she-camels. Throughout the current study, serum values of P4 and cortisol were higher than their reference ranges mentioned by Ayoub et al. (2003); Saeb et al. (2010), respectively, while serum E2 levels were within their reference values mentioned by Ayoub et al. (2003); Mohamed et al. (2021). Khalphallah et al. (2022) revealed that serum values of P4 and E2 were higher than their reference values on day 0 (Calving day) in she-camels either control or treated groups i.e. Selenium-vitamin E or multivitamin treated groups. Serum P4 levels were significantly reduced while serum E2 were greatly improved on day of calving (Day 0) in treated lactating she-camels comparing with their values in control lactating she-camels. Ayoub et al. (2003) reported that the values of sex steroid hormones were variable depending on various physiological circumstances whereas the pregnant she-camels had higher concentrations of sex steroid hormones than those in non-pregnant ones. Moreover, the source of highly reported serum P4 on day 0 post-calving in the examined lactating camels would be either adrenal or ovarian. On the other side, luteal activity that was detected even in non-mated she-camels in some cases due to spontaneous ovulation, was possibly induced (Elias 1990; Ayoub et al. 2003; 51). Mohamed et al. (2021) stated that serum levels of P_4_ were clearly lower while serum E_2_ levels were greater in nonpregnant she-camels than those in lactating ones. The pregnant she-camels had a significant upraise in serum cortisol values comparable with cortisol values in lactating or non-pregnant she-camels. No remarkable variations were revealed between lactating and non-pregnant animals. The highest levels of serum P_4_ and cortisol had been stated during pregnancy in investigated she-camels. As a stress indicator, the blood cortisol levels elevated following transportation (Anderson et al. 1999), New-world camelids hypoxemia (Riquelme et al. 1998). Higher concentrations of serum cortisol were reported on the day of parturition that could be owned to stress of parturition whereas it was associated with the foetal pituitary’s higher release of adrenocorticotropic hormone on the day of parturition (Tharwat et al. 2015; Ebissy et al. 2019). On the other side, Abdel-Rahman et al. (2017) reported significant elevations in blood cortisol values during the third trimester and this significant elevation persisted throughout the prepartum period, reaching their highest values on the day of parturition. Afterwards, a clear drop in their blood levels was reported during the few days following parturition up to 15 days postpartum.

#### Serum oxidants/antioxidants biomarkers

Abdel-Rahman et al. (2017) monitored the alterations in serum MDA levels during different stages of pregnancy and around calving whereas a significant raise in serum MDA levels was stated in the last trimester comparing with the other pregnancy stages and post-calving. They also added that serum concentrations of MDA were over than their reference ranges during she-camel gestation period. Referring to the present work, post-calving she-camels showed remarkable changes in serum oxidant/antioxidant biomarkers. Regarding oxidants indicators or biomarkers, concentrations of serum MDA were significantly higher in on day 14 after calving comparing with days 28 and 42 in post-partum she-camels. Furthermore, Serum MDA values were over than their reference ranges on days 14 and became within the reference values demonstrated by El-Bahr and El-Deeb (2016); Saleh et al. (2009); El-Deeb and Elmoslemany (2015), on days 28 and 42. Mohamed et al. (2021) confirmed significant drop in serum MDA in lactating or non-pregnant she-camels comparing with pregnant she-camels whereas serum values of MDA were higher than their reference ranges in pregnant she-camels, however, they were within their reference ranges in lactating and non-pregnant she-camels. Moreover, no significant alterations were demonstrated for serum MDA between lactating and non-pregnant groups. These results might be owned to physiological oxidative stress throughout pregnancy which induced overproduction of free radicals as well as lipid peroxidation (Abdel-Rahman et al. 2017). Walsh (1994) stated that lipid peroxidation happened naturally in cells and thus free radicals’ production was thought a normal physiological process, however, increased free radicals’ production during gestation could be due to the stressful condition that might cause several metabolic changes like elevated basal metabolic rates. This increase in free radicals induced an aggravation in the oxidative stress that associated with increased lipid peroxidation (Walsh 1994). The current study revealed higher levels of serum GSH on days 28 and 42 in post-partum she-camels than their values on day 14. On days 14 and 28 post-calving, serum levels of GSH in examined she-camels were lower than their reference ranges reported by Mohamed et al. (2021) then they reached their reference ranges on day 42 post-calving. Mohamed et al. (2021) added that with exception of SOD, serum levels of measured antioxidants indicators mainly serum GSH, were clearly lower in pregnant she-camels than lactating and non-pregnant ones. The previous studies interpreted that the low values of total antioxidants could be attributed to the adaptive capacity in order to optimize the oxygen consumption and neutralization of free radicals (Abdel-Rahman et al. 2017). On the other hand, earlier studies noted that CAT and GSH blood levels were noticeably lower in lactating and pregnant camels than in control camels. The most major antioxidant enzymes, GSH and CAT, catalyzed the conversion of superoxide anion into hydrogen peroxide and water, which inactivated significant amounts of oxidants. GSH was a key role in scavenging t-butyl hydroperoxide that was a substance that induced lipid peroxidation (Trotta et al. 1982; Mates 20001). Among these oxidants/antioxidants indicators, blood MDA values in both control and lactating camels were the same but GSH and CAT were clearly decreased in lactating camels where it stated that antioxidant defenses i.e. CAT and GSH, exhausted earlier than the raise in levels of blood lipid peroxidation i.e. MDA (Singh et al. 2013). Abdel-Rahman et al. (2017) reported significant decline in serum concentrations of TAC and GSH in the third trimester than others, hence, their prepartum levels were relatively lower than those at 15 days postpartum. Moreover, remarkable changes were reported by Mohamed et al. (2021) in serum levels of some antioxidant parameters i.e. TAC, CAT and GSH, between lactating, pregnant and non-pregnant groups whereas their serum values were significantly higher in non-pregnant she-camels than those in lactating group or in pregnant she-camels group. Moreover, serum values of GSH were greatly dropped in late stage of pregnancy then elevated gradually until early lactation onset. This could be attributed to the activity of blood GSH that was increased with elevated lipid peroxidation values, whereas serum GSH activity was negatively correlated with serum MDA production (8; Abdel-Rahman et al. 2017).

### Serum lipid profiles indicators

The current study reported remarkable changes in serum lipid profiles in post-calving she-camels whereas some of them i.e. glucose was significantly higher while some of them i.e. TPs, albumins and globulins, were significantly lower on day 14 than their values on days 28–42 in post-partum she-camels. on days 14–42 post-calving, serum concentrations of glucose, TPs, albumins and globulins in examined healthy she-camels were within the reference ranges reported by Mohamed and Hussein (1999); Kaneko et al. (2008); Saeb et al. (2010); Abdalmula et al. (2019); Islam et al. (2019). These results supported by Ebissy et al. (2019); Tharwat et al. (2015) whereas blood glucose levels had a significant progress in she-camels at calving time in comparison with their peripartum period values as this might be owned to calving as a stress and coincided with high blood cortisol levels during this period. On the other hand, Saeed et al. (2009); Kelanmer et al. (2018) reported a significant reduction in glucose concentration during periparturient period in dromedary camels comparing with the post-calving period. These findings could be the result of the gestational state’s impact on glucose levels. Moreover, Mohamed et al. (2021) reported no significant differences for blood glucose between lactating she-camels, pregnant she-camels and non-pregnant she-camels whereas they had serum blood concentrations within reference values. In contrast, a clear reduction in serum TPs and globulins were reported in lactating she-camels, pregnant she-camels comparing with non-pregnant she-camels. These significant variations were not stated for albumins between different animals’ groups. Omidi et al. (2015) agreed with Mohamed et al. (2021) results and they could be explained by the adequate homeostasis that could keep the blood glucose levels within a narrow range. Additionally, the sampling time did not vary according to the season (Omidi et al. 2015). To evaluate the health and physiological status of late pregnancy, parturition, and the postpartum periods in dromedary camels, it was required to estimate various serum parameters, including TPs, albumins, and globulins. In contrast to the current results, serum levels of TPs and albumins were not changed greatly either pre-calving or post-calving. However, globulin concentrations were remarkably raised on day 28 following parturition. This might be attributed to the formation of immunoglobulins (Tharwat et al. 2015; Ebissy et al. 2019). Moreover, plasma values of TPs and albumins were not altered as a result of the gestation period (Abd El-Hamid et al. 2021).

### The palatability of milk

Testing milk for sensory properties was also named organoleptic testing and uses the taste, smell and normal senses of sight to determine the overall acceptability. The result of this test was of minimum cost and obtained immediately (Dugassa 2021). In the present study the results revealed showed that the sensory properties of milk were improving over time. The color on day 14 seemed a little unfamiliar, then it improved on day 28 and became very normal on day 42, might be due to the disappearance of the dark-colored colostrum proteins. Likewise, the smell and taste improved and reached the usual limits on day 42, although the taste of natural camel milk was characterized by some salinity due to its high chlorine content (Khaskheli et al. 2005). Milk sensory properties were affected by physiological, genetic and multiple environmental factors. Furthermore, there were several physiological factors which had a great impact on organoleptic properties of milk including age at first calving, lactation stage, calving order and cow age. Generally, milk production generally increased when cows became older (Barreto et al. 2016).

## Milk oxidant/antioxidant biomarkers i.e. MDA and cortisol assays

A detrimental effect on milk and other dairy products was induced by oxidative processes as it caused shortening their shelf-life and retrograding their nutritional quality (Havemose et al. 2006; Krzyżewski et al. 2012). Higher antioxidant concentrations were associated with a delay or elimination of protein oxidation processes, according to Havemose et al. (2004). Antioxidant protection was supplied by appropriate meals and supplements. The amount of antioxidants protection in dairy products were determined using the molar ratio of antioxidants to oxidants (Cholesterol). Higher levels of antioxidant protection (DAP) enabled higher stability of dairy products by impacting their high antioxidative potential in response to lipid peroxidation, which caused detrimental changes in the nutritional content of milk and its products (Kapusta et al. 2018). The examined post-partum she-camels in the current work had significantly lower values of milk MDA as milk oxidant indicators on days 28 and 42 post-partum than their values on day 14. MDA values in she-camels milk were within their reference ranges mentioned by Kapusta et al. (2018). MDA as lipid peroxidation end products, was one of the most common reliable used indexes of oxidative stress. These findings might be attributed to the significant influence of milking performance on MDA levels in milk. The highest milk output was correlated with the highest MDA content. This confirmed the detrimental effects of an animal’s high milk yield on the quality of their milk (Kapusta et al. 2018). In case of high milk yield, large ruminants were more susceptible to free radicals as well as free radical peroxidation processes that induced MDA production (Sharma et al. 2011). Castillo et al. (2006) reported reducing milk MDA values after the peak of lactation was over as well as the metabolic status stabilized. Additionally, during early lactation or just after parturition, Sharma et al. (2011) confirmed that animals were more exposed to more oxidative stress and low antioxidant defense and this might explain their elevated susceptibility to production diseases including retention of fetal membranes, mastitis and metritis. Regarding to changes in milk cortisol levels via the present study, a significant drop in milk concentrations of cortisol as stress indicator was demonstrated on day 28–42 after calving in she-camels comparing with their values on day 14. Milk levels of cortisol was higher than their references ranges stated by Nedić et al. (2017) throughout the present work i.e. days 14–42 post-calving. The previous reports mentioned that lipid peroxidation was a process in which omega-3 and omega-6 fatty acids’ carbon double bonds were damaged by free radicals where this process end product was reactive aldehydes i.e. MDA. Moreover, Lipid peroxidation often induced adverse changes in the nutritional value of milk, hence, higher DAP could lead to higher stability of dairy products by improving their antioxidative efficacy (Kapusta et al. 2018).

### Milk SCC

Referring to SCC changes, SCC in milk had long been utilised as an indication of the severity of udder inflammation in cows and to forecast udder infection as well as subclinical mastitis (Poutrel and Rainard 1982). The references in camel were more recent (Kaskous 2019), therefore, with the exception of intramammary infections, the variation factors were not widely studied. Although, the highest levels had been recommended as 400 × 1000 cells/ml for cow milk by EU directives, this level was stated to be 250 × 1000 cells/ml for camels (Abbood 2016). At 14th day post-partum, the investigated she-camels in the current study had much higher values of Milk SCC then SCC was significantly dropped afterwards on days 28–42 following calving. Milk SCCs through the ongoing study were within the reference values stated by Saleh et al. (2013); Hamed et al. (2010). Khalphallah et al. (2022) supported these results whereas they mentioned significant elevations in milk SCC in control she-camels on day 14 comparing with those of days 21 and 28 post-partum. In contrast, the treated she-camels’ groups had lower values of milk SCC day 14 than those of days 21 and 28 post-partum. They also reported significant variations in milk SCC between control healthy camels and other treated post-calving she-camels’ groups i.e. days 14, 21 and 28. On other hand, other articles revealed that the unique composition of one humped camels milk considered one of one of the primary considerations for ranching camels. Moreover, losses in milk production could be reduced by decreasing SCC levels. Therefore, camel breeders were greatly advised by using strict hygienic measures, proper milking processes and regular subclinical mastitis test in their herds (Atasever and Koç 2016). Currently, several studies aimed to measure SCC of raw milk in camels (Wernery et al. 2008; Saleh and Faye 2011; Nagy et al. 2013). However, milk SCC levels had wide variations via several investigations. Furthermore, a lack of information about the association of milk production and losses with milk SCC values, had been reported (Atasever and Koç 2016).

### Correlations between serum steroids hormones, serum lipids, serum-milk oxidant/antioxidant indicators and milk SCC

Referring to the present results, different correlation relationships were reported between serum steroids hormones, serum lipid profile indices, serum oxidant/antioxidant indicators (Stress indicators) i.e. MDA and GSH and milk oxidant/antioxidant indicators (Stress indicators) i.e. MDA and cortisol, and milk SCC in clinically healthy post-calving she-camels.

No significant correlations were reported between serum steroid hormones i.e. P4, E2 and cortisol at post-calving period in examined she-camels. Serum P4 values were not correlated with lipid profiles indices i.e. glucose, TPs, albumins and globulins. Serum E2 were negatively correlated with each of serum glucose and globulins while blood cortisol values were correlated positively with glucose and negatively with blood proteins i.e. TPs, albumins and globulins. On the other hand, Mohamed et al. (2021) supported these results but serum E2 values were negatively correlated with TPs and globulins, however, they were not correlated with serum albumins or glucose. Serum cortisol in she-camels were not correlated with these lipid profiles indices. The present work revealed that she-camels at post-partum period (Days14-42) had positive correlations between steroids hormones i.e. P4 and cortisol, and serum MDA (Stress and oxidant indicators), hence, negative correlations were stated between serum GSH (Antioxidant indicators) and these steroid hormones (P4 and cortisol). No correlations were reported between E2 and each of serum MDA and serum GSH. In contrast, Mohamed et al. (2021) mentioned negative correlations between serum MDA and blood values of cortisol and P4, hence, serum E2 concentrations were a correlated positively with serum MDA levels. Blood E2 levels was correlated positively cortisol while negative correlations had been reported between serum levels of E2 and cortisol, and serum P4. On other hand, in she-camels with controlled-internal drug release (CIDR), re-used CIDR or OvSynch protocols, negative correlations were described between serum P4 and MDA in examined camels (Abo El-Maaty et al. 2019). The present study mentioned absence of significant correlations between serum steroids i.e. P4 and E2, and milk stress indictors i.e. MDA, cortisol and SCC. Serum cortisol levels were correlated positively with milk MDA, milk cortisol and SCC. In contrast Mohamed et al. (2021) mentioned that serum P4 levels were correlated positively with serum GSH. A negative correlation was stated between serum E2 and serum cortisol, and serum GSH. These correlations highly supported the finding of lower antioxidants and higher serum MDA in pregnant she-camels. Such a condition was probably associated with the reduced immune defense mechanism under high blood P4 values (Singh et al. 2013). Lactation status correlated positively with TAC, and negatively with P4 levels (Abd El-Hamid 2021).

#### **Correlations between serum lipids, serum-milk oxidant/antioxidant indicators and milk SCC in clinically healthy post-calved she-camels**

No correlations were stated in examined post-partum she-camels (Days 14–42) between blood glucose and each of other lipid profiles indices, serum oxidant/antioxidant biomarkers, milk oxidant/antioxidant biomarkers and milk SCC. Positive correlations were observed between serum proteins indicators i.e. TPs, albumins and globulins in investigated she-camels at post-partum period (Days 14–42). These serum proteins were correlated positively with serum GSH, while, they were negatively correlated with each of serum MDA, milk MDA, milk cortisol and milk SCC. Moreover, the previous articles reported that the change of TPs, albumins and globulins around parturition was attributed to the transfer of albumins and γ-globulins and TPs from circulation to the mammary tissues (Kupczyński and Chudoba-Drozdowska 2002).

#### Correlations between serum-milk oxidant/antioxidant indicators and milk SCC in clinically healthy post-calved she-camels

With except for serum GSH, the examined post-calving she-camels (Days 14–42) had positive correlations between serum-milk oxidant/antioxidant parameters and milk SCC. Serum MDA, milk MDA, milk cortisol and milk SCC were correlated positively with each other. These biomarkers i.e. serum MDA, milk cortisol and milk SCC, were negatively correlated with serum GSH.

## Conclusions

The study concluded the influence of stress as a result of lactation in post-partum period in recently calved she-camels and its relationship with reproductive cyclicity as well as changes in serum steroids, lipid profiles, serum-milk oxidant/antioxidants parameters and milk SCC that was reflected through significant elevations in serum levels of P4, E2, cortisol, MDA and glucose, and milk values of MDA, cortisol and SCC as well as significant drop in serum levels of GSH, TPs, albumins and globulins on day 14 post-calving comparing with their values particularly on day 42.

The study stated variable correlation relationships between reproductive cyclicity parameters, lipid profiles, serum-milk oxidant/antioxidants parameters and milk SCC.

## Data Availability

The datasets used and/or analyzed during the current study are available from the corresponding author on reasonable request. Data was also available after publishing in this journal.

## Abbreviations

CIDR: controlled-internal drug release
DAP: antioxidant protection
E2: estradiol
GSH: reduced glutathione
Hb: haemoglobin
MDA: malondialdehyde
PCV: packed cell volume
P4: progesterone
RBCs: red blood corpuscles
ROS: reactive oxygen species
SCC: somatic cell count
TBA: thiobarbituric acid
TLC: total leucocytic count
TPs: total proteins
